# Association between systemic inflammatory response index and glaucoma incidence from 2005 to 2008

**DOI:** 10.3389/fmed.2025.1542073

**Published:** 2025-02-04

**Authors:** Xiang Li, Yi Qing Sun, Xiao Dan Zhong, Zhi Jie Zhang, Jia Feng Tang, Zhan Yang Luo

**Affiliations:** ^1^Eye Institute and Affiliated Xiamen Eye Center, School of Medicine, Xiamen University, Xiamen, China; ^2^Chongqing Key Laboratory of Development and Utilization of Genuine Medicinal Materials in Three Gorges Reservoir Area, Chongqing Three Gorges Medical College, Wanzhou, China; ^3^Guangzhou University of Chinese Medicine, Guangzhou, China; ^4^Department of Pharmacy, Shanghai Pudong Hospital, Fudan University Pudong Medical Center, Shanghai, China

**Keywords:** systemic inflammatory response index, glaucoma, NHANES, systemic inflammation, biomarkers

## Abstract

**Objective:**

This study aimed to investigate the association between the Systemic Inflammatory Response Index (SIRI) and glaucoma using data from the 2005–2008 National Health and Nutrition Examination Survey (NHANES).

**Methods:**

We performed a cross-sectional analysis using data from NHANES (2005–2008). Among participants who underwent non-mydriatic retinal imaging and Frequency Doubling Technology (FDT) visual field testing, 4,514 were included after excluding those with missing key variable data. SIRI and other inflammatory indices, including the systemic immune-inflammation index (SII), platelet-to-lymphocyte ratio (PLR), and neutrophil-to-lymphocyte ratio (NLR), were calculated from blood samples. Logistic regression models were employed to assess the relationship between these indices and glaucoma, adjusting for demographic and health-related variables.

**Results:**

A significant positive association was found between elevated log_2_SIRI levels and the prevalence of glaucoma (Model 3: OR 1.24, 95% CI 1.07–1.44, *p* < 0.005). We performed an in-depth analysis of the Log_2_SIRI quartiles and found a significant association between Log_2_SIRI Q4 and the occurrence of glaucoma (Model 3: OR1.62, 95%CI 1.12–2.34, *p* = 0.011). This correlation was further validated using the area under the receiver operator characteristic curve (AUC) in Model 3(AUC = 0.674).

**Conclusion:**

Elevated SIRI levels are significantly associated with the prevalence of glaucoma, highlighting the potential role of systemic inflammation in glaucoma pathogenesis. SIRI may serve as a useful biomarker for identifying individuals at risk of glaucoma, facilitating early detection and targeted intervention strategies. Further research is needed to validate these findings and explore their clinical applications.

## Introduction

It is a chronic progressive condition characterized by the degeneration of retinal ganglion cells (RGCs) and their axons (1). Among individuals aged 40 to 80, this condition causes significant changes to the optic disc and visual field defects, making it the second leading cause of irreversible blindness worldwide (2). The number of people affected by glaucoma is projected to reach 111.8 million by 2040. The primary types of glaucoma include primary open-angle glaucoma (POAG), primary closed-angle glaucoma (PCAG), and normal tension glaucoma (NTG). Although the exact pathophysiology of glaucoma is not fully understood, elevated intraocular pressure (IOP) is recognized as a key risk factor for its progression (3). Glaucoma is a complex disorder influenced by various factors, including age, sex, hypertension, genetic variations, and environmental risks. Studies suggest that in POAG, inflammatory processes may directly link elevated IOP and ischemia to RGC degeneration. Inflammation typically responds to ischemic damage by producing pro-inflammatory substances and allowing various inflammatory cells to infiltrate ischemic tissues through gaps in the vascular endothelium. There is substantial evidence supporting a close link between inflammation and glaucoma (4, 5).

While inflammation is a natural process for tissue repair, uncontrolled inflammation can lead to tissue damage. To better assess an individual’s inflammatory status, new laboratory tests and indices have been developed (6–8). Evaluating inflammatory markers related to glaucoma has garnered significant interest. A number of studies have shown that inflammatory markers such as NLR and PLR are closely linked to adverse outcomes in cardiovascular diseases, cancer, and chronic kidney disease (9).

Neutrophils, monocytes, lymphocytes, and platelets are incorporated into the SII and the SIRI. These indices provide more comprehensive clinical insights than peripheral blood cell counts alone (10). Currently, no studies have investigated the association between SIRI and glaucoma (11).

NHANES is a multistage probability sampling method used by the Centers for Disease Control and Prevention (CDC) to represent the non-institutionalized U.S. population. Despite its widespread application in various research domains, the dataset has yet to be utilized to investigate the potential relationship between the SIRI and glaucoma (12).

## Materials and methods

### Subject selection and data source

For this study, we utilized publicly available NHANES data from 2005 to 2008. NHANES is a comprehensive and nationally representative cross-sectional survey of the non-institutionalized civilian population conducted by the National Center for Health Statistics (NCHS) under the CDC (13). It consists of detailed interviews, physical examinations either at home or in mobile examination centers (MECs), and laboratory tests. The survey is conducted biennially (14).

The NCHS Institutional Review Board approved the study, and all participants provided written informed consent. We chose the 2005–2008 data because it included relevant information on glaucoma status. Data were obtained from the publicly accessible NHANES database, with certain restricted data available upon limited access. We complied with all data usage regulations and anonymized personal information to ensure privacy.

**Figure 1:**
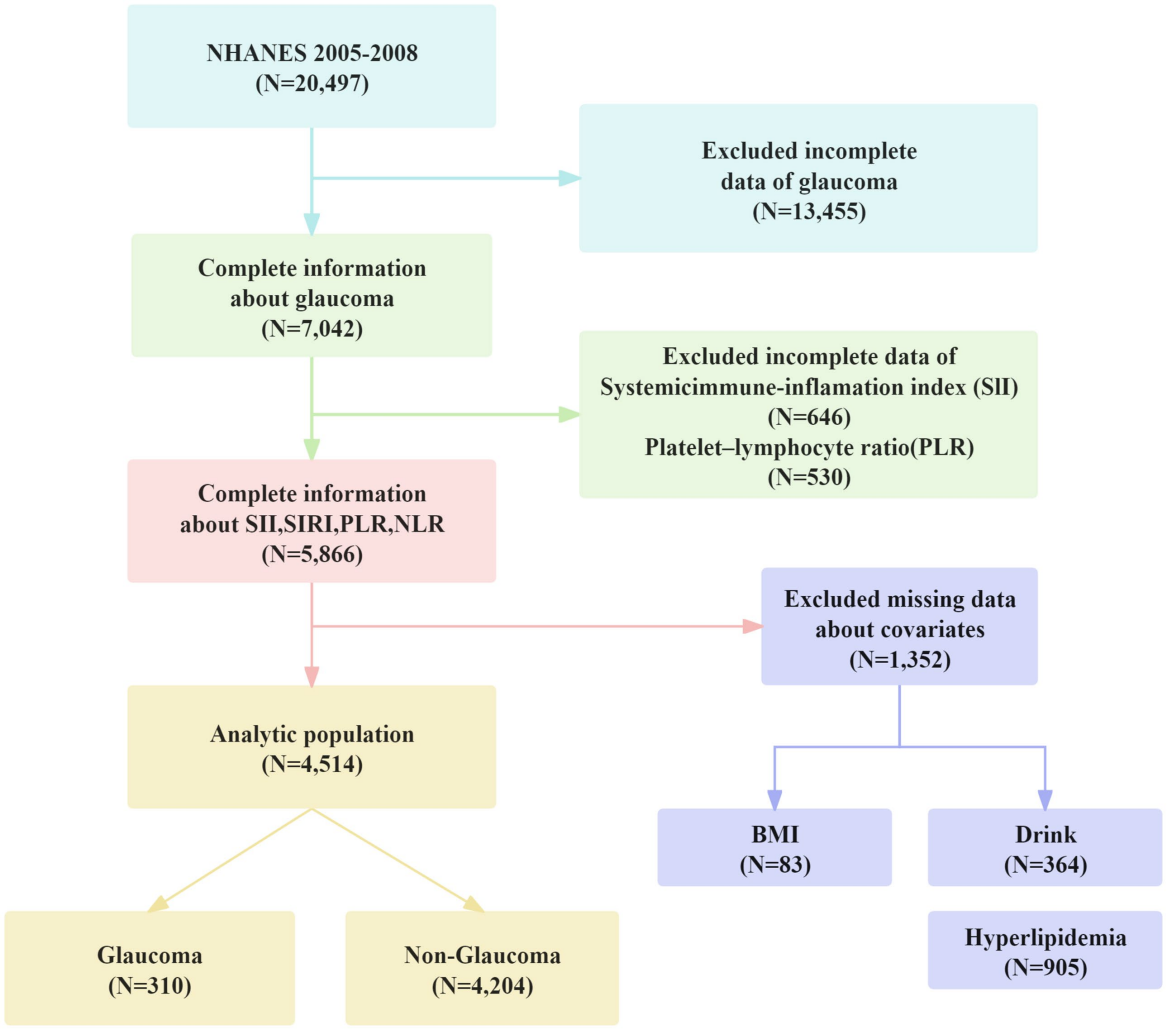
Screening process of the included studies.

In this study, individuals who did not have data on NLR, PLR, SII, SIRI, glaucoma status, and other essential covariates were excluded, resulting in a final sample size of 4,514 participants. The sample selection process is detailed in Figure 1. This approach ensured a nationally representative sample, enabling reliable analysis of the relationships between NLR, PLR, SII, SIRI, and glaucoma status.

### Defining criteria for glaucoma

Retinal imaging used a Canon CR6-45NM non-mydriatic camera to capture two 45° images per eye, focusing on the macula and optic nerve. These images were evaluated for vertical cup-to-disc ratio (vCDR) and asymmetry. In 2012, ophthalmologists re-evaluated images with a vCDR ≥0.6, categorizing them based on glaucoma-specific characteristics, with discrepancies resolved by consensus (15).

FDT testing, conducted by trained investigators using the Humphrey Matrix Visual Field Instrument, followed the N-30-5 protocol. An abnormal visual field was defined by at least two locations below the 1% threshold in both tests. Reliability checks included random assessments for false positives and blind spots (16).

Glaucoma diagnosis followed ISGEO and Rotterdam criteria, considering optic nerve appearance and glaucomatous visual field defects (GVFD).

Diagnosis criteria included:

CDR of any eye exceeding the 99.5th percentile of the NHANES population.
CDR asymmetry between eyes exceeding the 99.5th percentile (17).
CDR of any eye exceeding the 97.5th percentile with abnormal FDT results.
CDR asymmetry between eyes exceeding the 97.5th percentile with at least one eye showing abnormal FDT results (18).

### Inflammation-related index measurement

In the NHANES MEC, whole blood samples were analyzed following the procedures outlined in the NHANES Laboratory Procedures Manual (LPM), which specifies the methods for specimen collection and processing. This study focused on key inflammation-related indices: platelet count (PLT), neutrophil count (NC), lymphocyte count (LC), monocyte count (MC) and C-reactive protein (CRP). To explore the relationship between these indices and glaucoma, we calculated the SII, SIRI, PLR, and NLR. The PLT, NC, MC, and LC were measured in units of 1,000 cells/μL, while C-reactive protein was measured in mg/dL.


$$SII=\frac{PLT\times NC}{LC}$$

$$\mathrm{SIRI}=\frac{NC\times MC}{LC}$$

$$PLR=\frac{PLT}{LC}$$

$$NLR=\frac{NC}{LC}$$

### Covariates assessment

Demographic variables such as age, race/ethnicity, gender, education level, marital status, and Body Mass Index (BMI) were included as covariates in our study. This demographic data was gathered through computer-assisted personal interviews (19). Considering the established relationships between socio-economic status, living conditions, and health, these demographic factors were used to infer the participants’ social and living circumstances. Additional covariates included drinking status, smoking status, hypertension, hypercholesterolemia, and diabetes.

### Statistical methods

Data analysis was performed using *R*^2^ and EmpowerStats software from X&Y Solutions, Inc., Boston, MA, available at http://www.empowerstats.com. The analysis accounted for NHANES’ complex sampling design by incorporating sampling weights, strata, and primary sampling units. Continuous variables were presented as means ± standard errors (SE), while categorical variables were expressed as percentages ± SE. Chi-square tests or T-tests were used to examine demographic differences.

Due to right-skewed distributions of SII, SIRI, PLR, and NLR data, a natural logarithm transformation was applied for statistical analysis. Appropriate NHANES sampling weights were also utilized. Weighted logistic regression models were employed to evaluate the association between SII, SIRI, PLR, and NLR levels and glaucoma risk. Model 1 was unadjusted; Model 2 adjusted for age, race, gender, education, and marital status; and Model 3 further adjusted for smoking, alcohol consumption, BMI, hypertension, hypercholesterolemia, diabetes and CRP. These analyses revealed a significant association between SIRI levels and glaucoma occurrence. Weighted quantile regression analysis was used to further investigate these relationships. Forest plots visually represented the results of logistic regression, while smoothed curve fitting examined the approximately linear relationship between SIRI levels and glaucoma occurrence. Additionally, we assessed performance of predictive in the cohort using the area under the receiver operator characteristic curve (AUC). A *p*-value <0.05 was considered statistically significant. The data cleaning process is illustrated in Figure 1.

## Results

### Description of baseline information of the study sample

This study included a total of 4,514 participants, of whom 4,204 did not have glaucoma, while 310 were diagnosed with glaucoma following screening. Table 1 presents the demographic and clinical characteristics of all participants.

**Table 1:** Weighted demographic characteristics of all participants.

Variables	Non-Glaucoma	Glaucoma	*p*- value
N	4,204	310	
Age (years)	57.06 ± 11.76	66.43 ± 11.82	<0.0001
BMI (kg/m3)	29.27 ± 6.66	28.79 ± 6.06	0.302
CRP(mg/L)	0.46 ± 0.92	0.38 ± 0.54	0.206
SII	613.71 ± 354.46	651.12 ± 393.30	0.129
SIRI	1.27 ± 0.81	1.49 ± 0.96	0.0001
PLR	143.90 ± 56.89	150.32 ± 73.20	0.108
NLR	2.25 ± 1.09	2.47 ± 1.26	0.004
Gender,%			0.438
Male	45.88%	48.55%	
Female	54.12%	51.45%	
Race,%			0.022
Mexican	4.25%	3.66%	
Other Hispanic	2.85%	2.69%	
Non-Hispanic white	79.89%	74.38%	
Non-Hispanic black	8.65%	15.32%	
Other race	4.35%	3.95%	
Education,%			0.0002
Less than high school	5.89%	12.21%	
High school or above	94.11%	87.79%	
Marital Status,%			0.009
Married or living with partner	66.90%	58.44%	
Unmarried or other	33.10%	41.56%	
Drink,%			0.010
No	27.44%	35.37%	
Yes	72.56%	64.63%	
Hypertension,%			<0.0001
No	56.03%	41.35%	
Yes	43.97%	58.65%	
Hypercholesteremia,%			0.0005
No	51.35%	39.25%	
Yes	48.65%	60.75%	
Diabetes Mellitus,%			<0.0001
No	88.22%	75.81%	
Yes	11.78%	24.19%	
Smoke,%			0.038
No	50.92%	43.75%	
Yes	49.08%	56.25%	

Mean +/− SD for: Age BMI CRP SII SIRI PLR NLR. p value was calculated by weighted linear regression model. % for: Gender Race Education Marital Status Drink Hypertension Hypercholesteremia Diabetes Mellitus Smoke. *p*- value was calculated by weighted chi-square test. Weighted by: WTMEC2YR/2.

Those with glaucoma were generally older, more likely to be married or living with a partner, had higher educational attainment, and were predominantly female. In addition, a history of smoking or alcohol consumption was associated with an increased likelihood of glaucoma. Similarly, those diagnosed with hypertension, hyperlipidemia, or diabetes exhibited a higher incidence of glaucoma. As shown in Table 1, participants with glaucoma recorded higher SII, SIRI, PLR, and NLR scores, thereby supporting our initial hypothesis. However, C-reactive protein (CRP) did not have a strong association with glaucoma in our study. (*p* = 0.207).

### Association between SIRI and glaucoma

**Figure 2:**
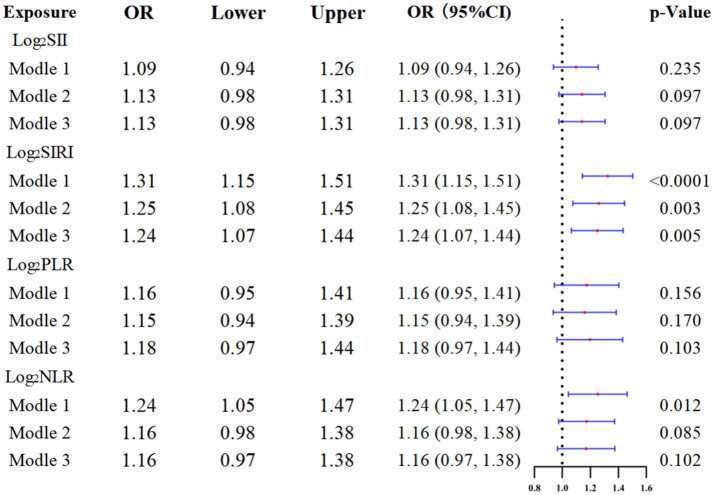
Forest plot of logistic regression results.

**Figure 3:**
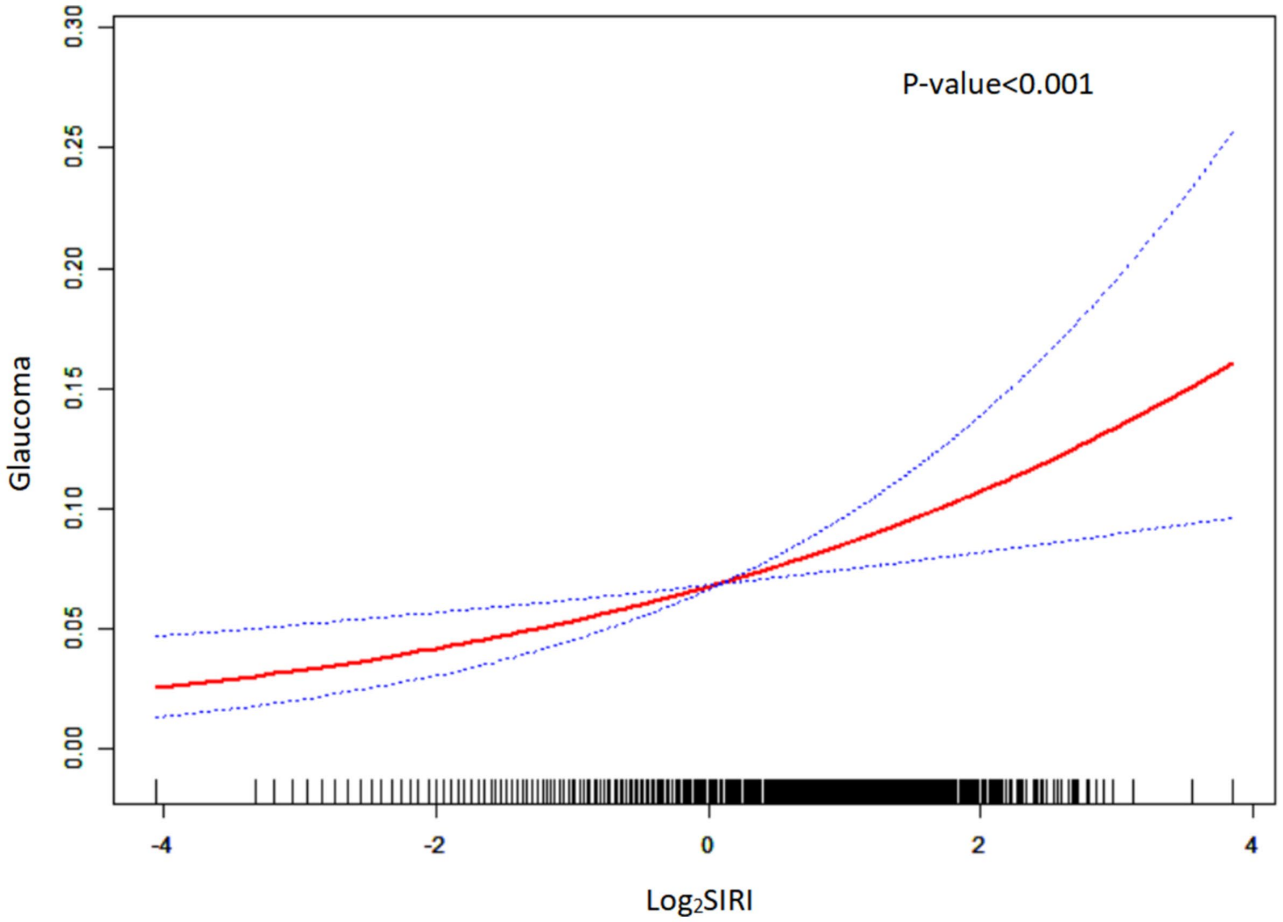
Smoothed curve fitting plot. The red solid line represents a smoothed curve fit of log_2_SIRI to glaucoma prevalence. The blue dashed line represents the 95% confidence interval of the smoothed curve fit.

The data of these indices exhibited a skewed distribution, so it was necessary to conduct a natural logarithm transformation during the statistical analysis. Table 2 presents the results of the multivariate regression analysis, while Figure 2 illustrates these findings, elucidating the relationships between various indices and glaucoma. In all models, a consistent positive correlation between log_2_SIRI and glaucoma was observed [Model 1: OR 1.31 (95% CI 1.15–1.51), *p* < 0.001; Model 2: OR 1.25 (95% CI 1.08–1.45), *p* = 0.003; Model 3: OR 1.24 (95% CI 1.07–1.44), *p* = 0.005]. Conversely, log_2_SII, log_2_NLR, and log_2_PLR did not show significant associations with glaucoma in Model 3. Furthermore, smoothed curve fitting, as illustrated in Figure 3, highlighted the approximately linear relationship (*p* < 0.001) between log_2_SIRI levels and glaucoma incidence, taking into account variables such as demographic factors, lifestyle habits, and health conditions.

**Table 2:** Weighted multivariate logistic analysis inflammatory markers and glaucoma.

Exposure	Model 1^a^ OR (95%CI)	*p*- value	Model 2^b^ OR (95%CI)	*p*- value	Model 3^c^OR (95%CI)	*p*- value
Log_2_SII	1.09 (0.94, 1.26)	0.235	1.13 (0.98, 1.31)	0.097	1.13 (0.98, 1.31)	0.097
Log_2_SIRI	1.31 (1.15, 1.51)	<0.0001	1.25 (1.08, 1.45)	0.003	1.24 (1.07, 1.44)	0.005
Log_2_PLR	1.16 (0.95, 1.41)	0.156	1.15 (0.94, 1.39)	0.170	1.18 (0.97, 1.44)	0.103
Log_2_NLR	1.24 (1.05, 1.47)	0.012	1.16 (0.98, 1.38)	0.085	1.16 (0.97, 1.38)	0.102

^aModel 1: a no adjusted. bModel 2: Gender; Age; Race; Education; Marital Status. cModel 3: Gender; Age; Race; Education; Marital Status; BMI; Drink; Hypertension; Hypercholesteremia; Diabetes Mellitus; CRP; Smoke.^

### Association between SIRI quartiles and glaucoma prevalence

**Figure 4:**
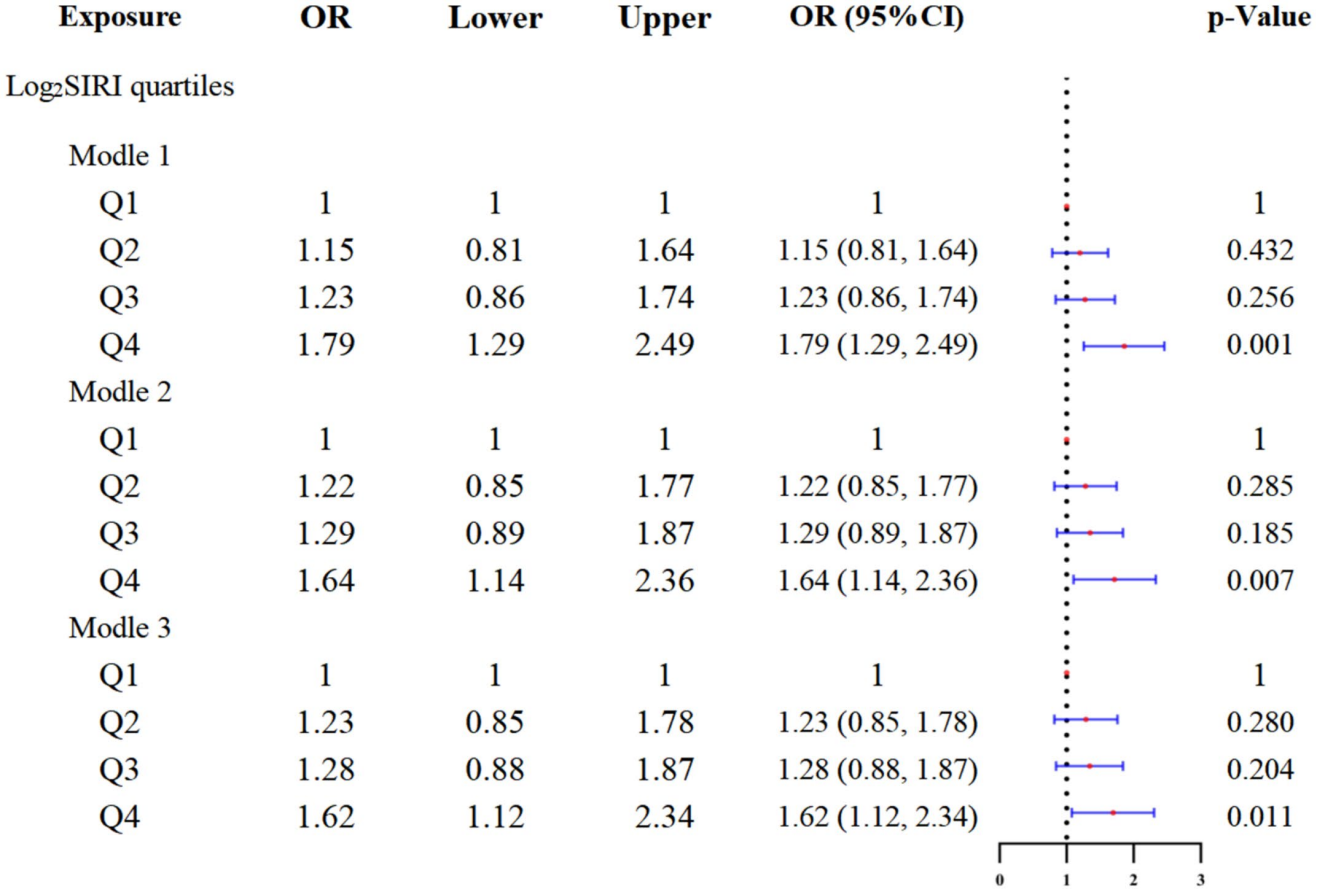
Forest plot of logistic regression results.

**Figure 5:**
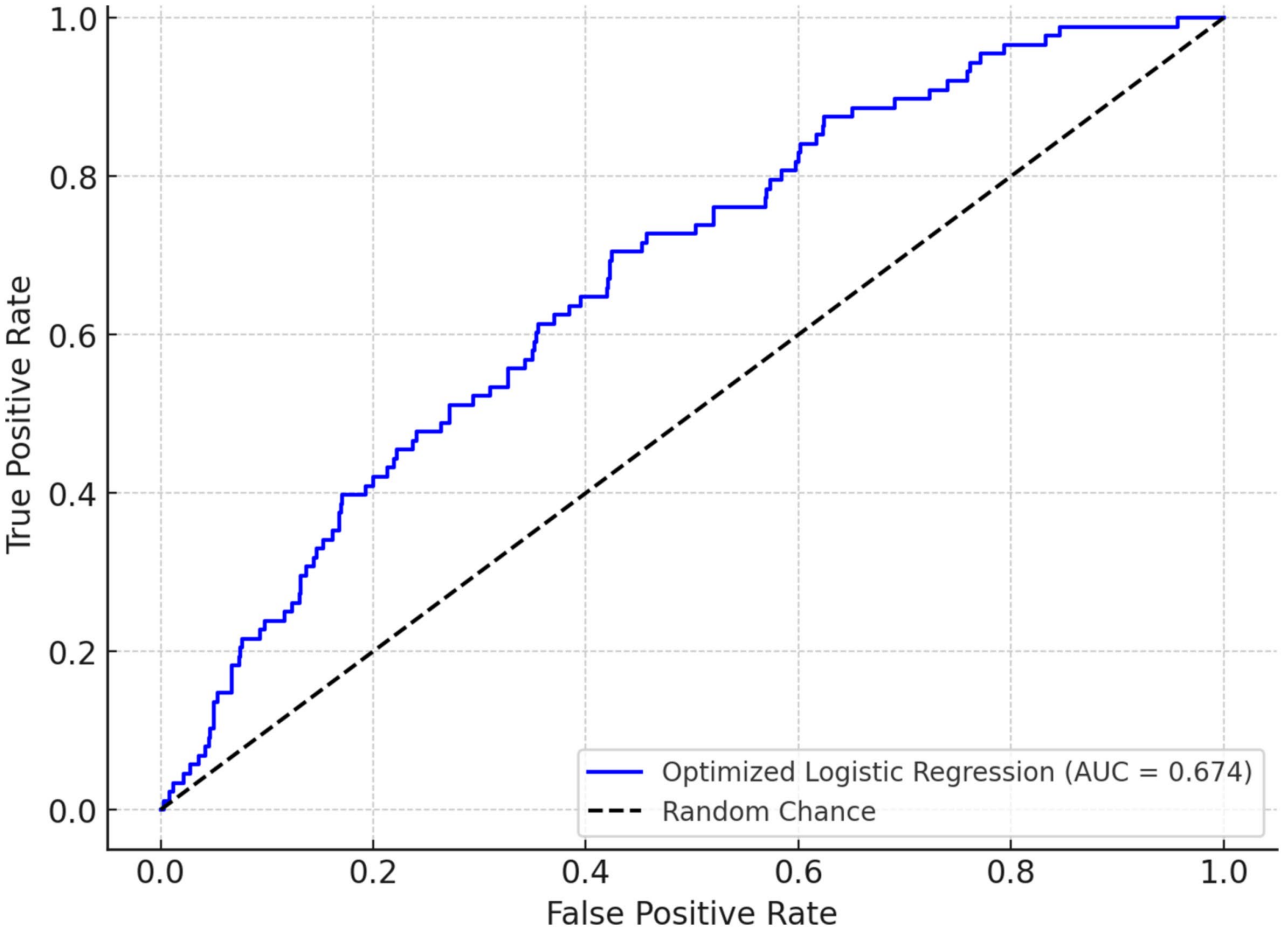
ROC Curve for Q4 of log_2_SIRI and glaucoma.

We divided the value of log_2_SIRI into four equal parts. Table 3 and Figure 4 explore the association between various log_2_SIRI quartiles and glaucoma prevalence. In the fully adjusted model (Model 3), the highest quartile (Q4) of log_2_SIRI showed a strong association with glaucoma (OR = 1.62, 95%CI = 1.12–2.34, *p* = 0.011). This positive correlation indicates that individuals in Q4 have a 62% higher risk of developing glaucoma compared to those in the lowest quartile (Q1). Additionally, the performance of Model 3 was evaluated using Q4 of log_2_SIRI, yielding an AUC of 0.674. This result indicates a significant association between Q4 of log_2_SIRI and the risk of glaucoma (Figure 5).

**Table 3:** Weighted multivariate logistic analysis systemic inflammatory response index and glaucoma.

Exposure	Model 1^a^ OR (95%CI)	*p*- value	Model 2^b^ OR (95%CI)	*p*- value	Model 3^c^OR (95%CI)	*p*- value
lnSIRI quartiles						
Q1	1		1		1	
Q2	1.15 (0.81, 1.64)	0.432	1.22 (0.85, 1.77)	0.285	1.23 (0.85, 1.78)	0.280
Q3	1.23 (0.86, 1.74)	0.256	1.29 (0.89, 1.87)	0.185	1.28 (0.88, 1.87)	0.204
Q4	1.79 (1.29, 2.49)	0.0005	1.64 (1.14, 2.36)	0.007	1.62 (1.12, 2.34)	0.011

^a^Model 1: a no adjusted. ^b^Model 2: Gender; Age; Race; Education; Marital Status. ^c^Model 3: Gender; Age; Race; Education; Marital. Status; BMI; Drink; Hypertension; Hypercholesteremia; Diabetes Mellitus; CRP; Smoke. Q1: [−4.06,−0.46]; Q2: [−0.46,0.1]; Q3: (0.1,0.6175]; Q4: [0.6175,3.85].

## Discussion

This study aimed to investigate the relationship between SIRI and glaucoma using data from the NHANES 2005–2008 dataset. Our results indicate a significant positive association between elevated SIRI levels and the prevalence of glaucoma. This association was particularly evident in specific subgroups, including women, older adults, and individuals with higher educational attainment.

The observed positive correlation between SIRI and glaucoma supports previous research indicating that systemic inflammation significantly contributes to the pathophysiology of glaucoma. Inflammatory processes are known to play a role in retinal ganglion cell damage and optic nerve degeneration, which are characteristic features of glaucoma. SIRI, encompassing neutrophil, monocyte, and lymphocyte counts, may act as a comprehensive marker of inflammatory status, reflecting both innate and adaptive immune responses (20–23).

We found that the association between SIRI and glaucoma was more pronounced in women. This gender difference might be due to hormonal influences on immune function and inflammatory processes. For instance, estrogens are known to modulate immune responses, which could impact susceptibility to inflammatory conditions such as glaucoma (21, 22). Additionally, older adults (aged 68–85) and individuals with higher education levels also showed a stronger association, indicating that age-related immune changes and socioeconomic factors might influence the relationship between systemic inflammation and glaucoma (20–24). We hypothesize that the relationship between systemic inflammation and glaucoma is notably stronger among individuals with higher educational attainment for several reasons. First, individuals with higher education typically pay closer attention to their health, undergoing regular check-ups and screenings, thereby increasing the likelihood of detecting subclinical inflammation or early-stage glaucoma (25). Second, academic or occupational pressures associated with higher education may result in elevated chronic stress levels, which can in turn exacerbate inflammatory responses (26). Third, potential confounding factors cannot be ignored: higher educational levels often correlate with higher income, better access to healthcare, and healthier lifestyles, all of which may interact with inflammatory biomarkers and glaucoma risk. Finally, considering that individuals with higher education may be more inclined to participate in health surveys, selection bias within NHANES database warrants attention (27). Such bias could lead to an overrepresentation of highly educated participants and, consequently, influence the observed relationship between educational attainment and glaucoma.

While the relationship between inflammation and glaucoma has been studied previously, our research is among the first to specifically investigate SIRI as a potential biomarker. Earlier studies mainly focused on other inflammatory indices such as the NLR and PLR, which also showed associations with glaucoma but were less comprehensive than SIRI (20–24, 28). In a study by K. Atalay et al., no significant differences in CRP levels were observed between patients with pseudoexfoliation and primary open-angle glaucoma and those in the control group (29). In a population-based prospective study, Simone de Voogd et al. further reported that serum CRP levels are not a significant risk factor for OAG (30). Additionally, research by Nurşen Yüksel et al. showed no differences in CRP levels among XFS, XFG, and control groups, and a meta-analysis investigating the association between high-sensitivity C-reactive protein and glaucoma concluded that there is no correlation between CRP and glaucoma (31). Consistent with these findings, our results also indicate no significant association (*p* = 0.206) between CRP and the prevalence of glaucoma. Our findings add to the growing evidence supporting the role of systemic inflammation in glaucoma and underscore the potential clinical utility of SIRI.

The performance of Model 3 was evaluated using Q4 of log2SIRI, yielding an AUC of 0.674. This result indicates a significant association between Q4 of log2SIRI and the risk of glaucoma. Identifying SIRI as a significant marker associated with glaucoma has important clinical implications. First, it offers a readily accessible and cost-effective tool for assessing systemic inflammation in patients at risk for glaucoma, which could improve early detection and intervention strategies, potentially slowing disease progression (32–34). Second, monitoring SIRI levels in glaucoma patients might help tailor anti-inflammatory treatments and evaluate their effectiveness (23).

Despite the strengths of our study, including the use of a large, nationally representative dataset and comprehensive adjustment for potential confounders, several limitations should be acknowledged. The cross-sectional design precludes causal inferences, and reliance on self-reported glaucoma diagnoses may introduce misclassification bias. Due to the limitations of the NHANES database, this study lacks a detailed classification of glaucoma, and because glaucoma is not a monolithic disease, the absence of subtype differentiation may overlook key differences in pathogenesis, disease progression, and responses to other risk factors among different types of glaucoma. Since we are unable to subdivide the glaucoma subtypes, our results can only represent the overall diagnosis of “glaucoma,” which may underestimate or overestimate the relevant factors or risk characteristics of certain specific subtypes. We call on future epidemiological surveys or large-scale databases to record information about different glaucoma subtypes more comprehensively, or to further subdivide glaucoma through other means, in order to more accurately assess the disease characteristics of patients with various subtypes and their relationships with other factors. Future studies should incorporate both clinical research and longitudinal designs to thoroughly investigate the relationship between different types of glaucoma and SIRI, as well as to establish potential causal links. While SIRI is a robust marker of inflammation, it does not capture all aspects of the immune response; therefore, exploring a broader range of biomarkers may provide a more comprehensive understanding. Furthermore, examining the impact of anti-inflammatory interventions on SIRI levels and glaucoma outcomes could deepen our insight into the role of systemic inflammation in glaucoma, ultimately guiding the development of targeted treatment strategies (24, 33–35).

## Conclusion

In conclusion, our study reveals a notable positive association between SIRI and glaucoma. These results highlight the role of systemic inflammation in the development of glaucoma and suggest that SIRI could be an effective biomarker for identifying and managing patients at risk. Further research is needed to validate these findings and investigate their potential clinical applications.

## Data Availability

Publicly available datasets were analyzed in this study. This data can be found at: https://www.cdc.gov/nchs/nhanes/index.htm.

## Glossary

SIRI: Systemic inflammatory response index
NHANES: National health and nutrition examination survey
FDT: Frequency doubling technology
SII: Systemic immune-inflammation index
PLR: Platelet-lymphocyte ratio
NLR: Neutrophil-lymphocyte ratio
RGC: Retinal ganglion cell
POAG: Primary open-angle glaucoma
PCAG: Primary closed-angle glaucoma
NTG: Normal tension glaucoma
IOP: Intraocular pressure
CDC: Centers for Disease Control and Prevention
NCHS: National Center for Health Statistics
MECs: Mobile examination centers
vCDR: Vertical cup-to-disc ratio
GVFD: Glaucomatous visual field defects
LPM: Laboratory Procedures Manual
PLT: Platelet count
NC: Neutrophil count
LC: Lymphocyte count
MC: Monocyte count
BMI: Body Mass Index
SE: Standard errors

## References

[1] Agarwal R Gupta SK Agarwal P Saxena R Agrawal SS. Current concepts in the pathophysiology of Glaucoma. Indian J Ophthalmol. (2009) 57:257–66. doi: 10.4103/0301-4738.53049, PMID: 19574692 PMC2712693

[2] Jonas JB Aung T Bourne RR Bron AM Ritch R Panda-Jonas S. Glaucoma. Lancet. (2017) 390:2183–93. doi: 10.1016/S0140-6736(17)31469-1 28577860

[3] Kang JM Tanna AP. Glaucoma. Med Clin North Am. (2021) 105:493–510. doi: 10.1016/j.mcna.2021.01.004 33926643

[4] Choi S Choi S-H Bastola T Park Y Oh J Kim K-Y. AIBP: a new safeguard against glaucomatous Neuroinflammation. Cells. (2024) 13:198. doi: 10.3390/cells13020198, PMID: 38275823 PMC10814024

[5] De Voogd S Ikram MK Wolfs RCW Jansonius NM Hofman A de Jong PTVM. Incidence of open-angle Glaucoma in a general elderly population: the Rotterdam study. Ophthalmology. (2005) 112:1487–93. doi: 10.1016/j.ophtha.2005.04.018, PMID: 16039716

[6] Chen Y Lin Y Vithana EN Jia L Zuo X Wong TY. Common variants near ABCA1 and in PMM2 are associated with primary open-angle Glaucoma. Nat Genet. (2014) 46:1115–9. doi: 10.1038/ng.3078 25173107

[7] Kim KE Kim MJ Park KH Jeoung JW Kim SH Kim CY. Epidemiologic survey Committee of the Korean Ophthalmological Society Prevalence, awareness, and risk factors of primary open-angle Glaucoma: Korea National Health and nutrition examination survey 2008-2011. Ophthalmology. (2016) 123:532–41. doi: 10.1016/j.ophtha.2015.11.004, PMID: 26746594

[8] Zukerman R Harris A Oddone F Siesky B Verticchio Vercellin A Ciulla TA. Glaucoma heritability: molecular mechanisms of disease. Genes (Basel). (2021) 12:1135. doi: 10.3390/genes12081135, PMID: 34440309 PMC8391305

[9] Vohra R Tsai JC Kolko M. The role of inflammation in the pathogenesis of Glaucoma. Surv Ophthalmol. (2013) 58:311–20. doi: 10.1016/j.survophthal.2012.08.010, PMID: 23768921

[10] Cheng W Bu X Xu C Wen G Kong F Pan H. Higher systemic immune-inflammation index and systemic inflammation response index levels are associated with stroke prevalence in the asthmatic population: a cross-sectional analysis of the NHANES 1999-2018. Front Immunol. (2023) 14:1191130. doi: 10.3389/fimmu.2023.1191130, PMID: 37600830 PMC10436559

[11] Erdogan T. Role of systemic immune-inflammation index in asthma and NSAID-exacerbated respiratory disease. Clin Respir J. (2021) 15:400–5. doi: 10.1111/crj.13314 33249745

[12] Hartwell ML Khojasteh J Wetherill MS Croff JM Wheeler D. Using structural equation modeling to examine the influence of social, behavioral, and nutritional variables on health outcomes based on NHANES data: addressing complex design, nonnormally distributed variables, and missing information. Curr Dev Nutr. (2019) 3:10. doi: 10.1093/cdn/nzz010, PMID: 31008441 PMC6465451

[13] Huang Z. Association between blood Lead level with high blood pressure in US (NHANES 1999-2018). Front Public Health. (2022) 10:836357. doi: 10.3389/fpubh.2022.836357, PMID: 35548077 PMC9081331

[14] Wu M Si J Liu Y Kang L Xu B. Association between composite dietary antioxidant index and hypertension: insights from NHANES. Clin Exp Hypertens. (2023) 45:2233712. doi: 10.1080/10641963.2023.2233712, PMID: 37439538

[15] Qiu M Boland MV Ramulu PY. Cup-to-disc ratio asymmetry in U.S. adults: prevalence and association with Glaucoma in the 2005-2008 National Health and nutrition examination survey. Ophthalmology. (2017) 124:1229–36. doi: 10.1016/j.ophtha.2017.03.049, PMID: 28545734

[16] Zhang Y Zhao Z Ma Q Li K Zhao X Jia Z. Association between dietary calcium, potassium, and magnesium consumption and Glaucoma. PLoS One. (2023) 18:e0292883. doi: 10.1371/journal.pone.0292883, PMID: 37851631 PMC10584168

[17] Boland MV Gupta P Ko F Zhao D Guallar E Friedman DS. Evaluation of frequency-doubling technology Perimetry as a means of screening for Glaucoma and other eye diseases using the National Health and nutrition examination survey. JAMA Ophthalmol. (2016) 134:57–62. doi: 10.1001/jamaophthalmol.2015.4459, PMID: 26562502

[18] Taechameekietichai T Chansangpetch S Peerawaranun P Lin SC. Association between daily niacin intake and Glaucoma: National Health and nutrition examination survey. Nutrients. (2021) 13:4263. doi: 10.3390/nu13124263, PMID: 34959814 PMC8709149

[19] Mahemuti N Jing X Zhang N Liu C Li C Cui Z. Association between systemic immunity-inflammation index and hyperlipidemia: a population-based study from the NHANES (2015-2020). Nutrients. (2023) 15:1177. doi: 10.3390/nu15051177, PMID: 36904176 PMC10004774

[20] Guo J Huang Y Pang L Zhou Y Yuan J Zhou B. Association of Systemic Inflammatory Response Index with ST segment elevation myocardial infarction and degree of coronary stenosis: a cross-sectional study. BMC Cardiovasc Disord. (2024) 24:98. doi: 10.1186/s12872-024-03751-z, PMID: 38336634 PMC10858502

[21] Huang Y-W Zhang Y Feng C An Y-H Li Z-P Yin X-S. Systemic inflammation response index as a clinical outcome evaluating tool and prognostic Indicator for hospitalized stroke patients: a systematic review and Meta-analysis. Eur J Med Res. (2023) 28:474. doi: 10.1186/s40001-023-01446-3, PMID: 37915088 PMC10621190

[22] Wang X Ni Q Wang J Wu S Chen P Xing D. Systemic inflammation response index is a promising prognostic marker in elderly patients with heart failure: a retrospective cohort study. Front Cardiovasc Med. (2022) 9:871031. doi: 10.3389/fcvm.2022.871031, PMID: 35911534 PMC9330028

[23] Zhang S Tang Z. Prognostic and Clinicopathological significance of systemic inflammation response index in patients with hepatocellular carcinoma: a systematic review and Meta-analysis. Front Immunol. (2024) 15:1291840. doi: 10.3389/fimmu.2024.1291840, PMID: 38469315 PMC10925676

[24] Luo S Liu Z Jiao R Li W Sun J Ma S. The associations of two novel inflammation indexes, systemic immune-inflammation index (SII) and system inflammation response index (SIRI), with periodontitis: evidence from NHANES 2009-2014. Clin Oral Investig. (2024) 28:129. doi: 10.1007/s00784-024-05529-1, PMID: 38300315

[25] Jayaram H Kolko M Friedman DS Gazzard G. Glaucoma: now and beyond. Lancet. (2023) 402:1788–801. doi: 10.1016/S0140-6736(23)01289-8, PMID: 37742700

[26] Saini C Davies EC Ung L Chodosh J Ciolino JB Jurkunas UV. Incidence and risk factors for Glaucoma development and progression after corneal transplantation. Eye (Lond). (2023) 37:2117–25. doi: 10.1038/s41433-022-02299-6, PMID: 36329167 PMC10333209

[27] Liu Z Hu Y Wang Y Xu B Zhao J Yu Z. Relationship between high dose intake of vitamin B12 and Glaucoma: evidence from NHANES 2005-2008 among United States adults. Front Nutr. (2023) 10:1130032. doi: 10.3389/fnut.2023.1130032, PMID: 37139451 PMC10149911

[28] Wei C-J Xue J-J Zhou X Xia X-S Li X. Systemic immune-inflammation index is a prognostic predictor for patients with acute ischemic stroke treated with intravenous thrombolysis. Neurologist. (2024) 29:22–30. doi: 10.1097/NRL.0000000000000508, PMID: 37582611

[29] Atalay K Savur FG Kirgiz A Kaldırım HE Zengi O. Serum levels of thyroid hormone, vitamin D, vitamin B12, folic acid, C-reactive protein, and hemoglobin in Pseudoexfoliation and primary open angle Glaucoma. J Fr Ophtalmol. (2019) 42:730–8. doi: 10.1016/j.jfo.2019.01.002, PMID: 31103354

[30] De Voogd S Wolfs RCW Jansonius NM Witteman JCM Hofman A de Jong PTVM. Atherosclerosis, C-reactive protein, and risk for open-angle Glaucoma: the Rotterdam study. Invest Ophthalmol Vis Sci. (2006) 47:3772–6. doi: 10.1167/iovs.05-1278, PMID: 16936085

[31] Al-Namaeh M. A meta-analysis of the association between high-sensitivity c-reactive protein level and Glaucoma. Eur J Ophthalmol. (2025) 35:29–39. doi: 10.1177/11206721241248019 38751132

[32] Chao B Ju X Zhang L Xu X Zhao Y. A novel prognostic marker systemic inflammation response index (SIRI) for operable cervical Cancer patients. Front Oncol. (2020) 10:766. doi: 10.3389/fonc.2020.00766, PMID: 32477958 PMC7237698

[33] Zhang C Li M Liu L Deng L Yulei X Zhong Y. Systemic immune-inflammation index as a novel predictor of major adverse cardiovascular events in patients undergoing percutaneous coronary intervention: a meta-analysis of cohort studies. BMC Cardiovasc Disord. (2024) 24:189. doi: 10.1186/s12872-024-03849-4, PMID: 38561664 PMC10985984

[34] Ding Y Liu Z Li J Niu W Li C Yu B. Predictive effect of the systemic inflammation response index (SIRI) on the efficacy and prognosis of Neoadjuvant Chemoradiotherapy in patients with locally advanced rectal Cancer. BMC Surg. (2024) 24:89. doi: 10.1186/s12893-024-02384-5, PMID: 38481180 PMC10935841

[35] Jin N Huang L Hong J Zhao X Hu J Wang S. The association between systemic inflammation markers and the prevalence of hypertension. BMC Cardiovasc Disord. (2023) 23:615. doi: 10.1186/s12872-023-03661-6, PMID: 38097927 PMC10720087

